# Diagnostic Accuracy of the Cincinnati Prehospital Stroke Scale in an Urban Emergency Department in Ghana

**DOI:** 10.7759/cureus.86991

**Published:** 2025-06-29

**Authors:** Hussein A Yakubu, Lawrence N Nsoh, Augustine K Sampah, Sandra Ofori, Ebenezer T Yeboah, Kwaku K Offe, Daniel Osei-Kwame

**Affiliations:** 1 Department of Emergency Medicine, Komfo Anokye Teaching Hospital, Kumasi, GHA

**Keywords:** acute stroke, cpss, emergency department, ghana, low-resource setting, prehospital screening tool

## Abstract

Background

The Cincinnati Prehospital Stroke Scale (CPSS) is a quick and easy-to-use tool that has been validated for early stroke identification, particularly in prehospital settings. Its utilization in low-resource settings, where access to imaging may be limited or costly, has not been thoroughly explored. This study sought to evaluate the diagnostic accuracy of the CPSS in a large urban emergency department (ED) in Ghana, which has a high volume of stroke admissions.

Methods

A prospective cross-sectional study was conducted in the adult ED at Komfo Anokye Teaching Hospital (KATH), Ghana’s second largest hospital. Patients, 18 years or older, who were referred or self-reported to KATH ED with clinical features suggestive of stroke, were identified by trained triage nurses upon arrival. Following informed consent, the nurses collected demographic information and also administered the CPSS. The components of the CPSS, which include facial droop, arm drift, and speech impairment, were each assigned a score of 1 if present and 0 if absent. All responses, including the total CPSS score, were documented on pre-designed paper forms and later entered into an electronic platform. A total score of ≥1 indicated a possible stroke. Patients were then followed throughout their hospital stay until they received brain computed tomography (CT) imaging, and the results interpreted by a specialist radiologist to confirm the diagnosis of stroke.

Results

A total of 110 individuals met the inclusion criteria and gave consent to participate. These included 51 males (46.4%) and 59 females (53.6%), with ages ranging from 33 to 92 years (median: 54 years, IQR: 48-67). The majority of patients (89, 89.9%) were referred. Eleven patients (10%) were unable to undergo CT imaging, while 99 patients (90%) completed CT imaging, with 86 (78.2%) having a confirmed stroke diagnosis. Upon reviewing the CPSS scores, 18 patients had incomplete entries and were excluded. The remaining 92 were analyzed. Among them, 35 patients (38.0%) scored 1 for face droop, 72 (78.3%) scored 1 for arm drift, and 72 (78.3%) scored 1 for speech impairment. Eighty-seven patients (94.6%) had a total CPSS score of ≥1. Sixty-nine patients (75.0%) had both a CPSS score of ≥1 and a confirmed stroke on CT scan. The CPSS had a sensitivity of 88.5% (CI: 69.8-97.6), a specificity of 25.5% (CI: 15.8-38.0), a positive predictive value (PPV) of 31.9% (CI: 21.4-44.0), and a negative predictive value (NPV) of 85.0% (CI: 62.1-96.8). Individually, face droop had the highest sensitivity (82.9%, CI: 66.4-93.4).

Conclusion

The CPSS, administered by trained triage nurses, showed a high sensitivity but low specificity in identifying patients with stroke at the KATH ED. It can serve as a valuable prehospital screening tool in low-resource settings. Future studies should explore its feasibility and adaptation across a wider group of prehospital personnel, including emergency medical technicians (EMTs) in Ghana.

## Introduction

Stroke remains a leading cause of death and disability globally, with a current burden that is heavily skewed towards lower- and middle-income countries where resources for treatment are constrained [1]. Early diagnosis of this disease is required for prompt intervention and favorable outcomes. Consequently, several tools have been designed to facilitate timely stroke identification, especially in the prehospital setting [2]. One of the most widely used is the Cincinnati Prehospital Stroke Scale (CPSS).

The CPSS was first designed in 1997 by the University of Cincinnati Medical Center for prehospital detection of stroke and has since been validated and adopted by many countries outside the United States, showing good sensitivity and accuracy for diagnosing strokes [3]. The CPSS is a three-item scale derived from a simplification of the National Institute of Health (NIH) Stroke Scale [4]. The components of the CPSS include arm drift, facial droop, and abnormal speech, each of which is scored 1 if present and 0 if absent. A total CPSS score ≥1 is required for a substantive stroke diagnosis with a probability of >72% [5]. If all three components are present, the likelihood of stroke is ≥85% [5].

The CPSS is primarily utilized in the prehospital setting, aiding paramedics to make early determinations of stroke and activating stroke care protocols, as well as deciding where to transport the patients [6]. It has also proven to be useful in patient triaging, as it can be used to quickly identify patients who could have a stroke and require early evaluation and imaging in the emergency room [7]. In settings where these imaging modalities are not readily available or may be delayed, the CPSS can accurately screen patients who need urgent attention and should be prioritized for imaging.

The accuracy and utility of the CPSS have not been well studied in low-resource settings, where stroke awareness is limited, patient presentations are delayed, and access to healthcare infrastructure can be challenging. Ghana, a lower-middle-income country in West Africa, faces similar challenges and currently has no accepted stroke screening tool. Additionally, the potential benefits of implementing such a tool in this context have not been thoroughly investigated.

The primary objective of this study was to evaluate the diagnostic accuracy of the CPSS in detecting stroke among patients presenting to the emergency department (ED) at Komfo Anokye Teaching Hospital (KATH). The secondary objective was to assess the feasibility of implementing the CPSS in a low-resource, urban emergency setting by trained triage nurses.

## Materials and methods

This prospective cross-sectional survey was conducted at the adult ED in KATH between November 2022 and March 2023. The study was approved by the KATH Institutional Review Board. KATH is a tertiary referral hospital located in the Ashanti region of Ghana, serving a population of 5.4 million people [8]. The hospital has a 37-bed adult ED that treats approximately 22,000 patients annually and receives the highest number of stroke referrals in central Ghana. The ED is equipped with a computed tomography (CT) scan, which serves as the primary imaging modality for stroke at this facility. All brain CT scans were interpreted by specialist radiologists, who provided formal reports that were uploaded to patients’ medical records. In this facility, patients were required to pay before undergoing a CT scan. All patients presenting to the ED were triaged using the South African Triage Scale (SATS) [9].

This study included patients aged 18 years or older who either self-reported or were referred with clinical features suggestive of stroke and gave consent for participation. Stroke clinical features were defined as signs and symptoms of neurological deficits that had a sudden onset or progressed rapidly, were focal or global, and were suspected to be of vascular origin [10]. Patients who arrived in a critically ill state, for instance, cardiorespiratory arrest or peri-arrest, and required immediate resuscitation were considered too ill to participate and were not approached. Additionally, patients younger than 18 years and those who refused consent were excluded.

Consecutive patients who met the inclusion criteria were identified upon arrival at the triage unit of the KATH ED by triage nurses. These nurses underwent a two-day training on how to identify prospective participants, obtain informed consent, and use the CPSS. Their training included practical demonstrations on how to ask questions uniformly, in both English and the predominant local language, ‘Twi’. Additionally, there were three days of pilot data collection, which allowed for the identification and correction of procedural errors, addressed study-related questions, and helped standardize the study process before recruitment began.

After triaging, prospective patients or their caregivers were approached for written consent before starting data collection. Using the CPSS, patients were asked to smile, elevate both arms forward for 10 seconds, and repeat a short phrase while the evaluator observed and assessed for facial droop/deviation, arm drift, or speech impairment. Caregivers were also asked if they had observed these symptoms (including new facial deviation, limb weakness/gait changes, or speech slurring) before arrival, especially for patients who were unresponsive in the ED, to corroborate the information.

All responses were recorded on pre-designed paper forms that were stationed at triage. Each of the CPSS components was scored 1 if present and 0 if absent. The total score was calculated and recorded on the forms, which were then signed and filed by the triage nurses. Additionally, data on sociodemographic characteristics, triage (SATS) category, referral status, and mode of arrival were collected. At the end of each day, the completed forms were submitted to research assistants who entered the information into an electronic platform using tablet computers.

All recruited patients were followed throughout their admission until they underwent brain CT imaging, and the scans were interpreted by a specialist radiologist to confirm the diagnosis of stroke. For patients who had already received CT imaging before arrival, the study team ensured that the scans were reported by a specialist radiologist before documenting the findings. These radiologists were unaware of the study and could not have been influenced in their reporting.

Research assistants also served as quality assurance officers and regularly followed up on data collection at triage to ensure consistency in data entry and the completeness of information, thereby minimizing errors. When scores could not be assigned, triage nurses were encouraged to document the reason on the form.

Data were analyzed using Stata Version 16.0 (StataCorp LLC, College Station, TX, USA). Descriptive analysis of all recruited patients was presented in tables and figures as frequencies and percentages. Patients with both complete CPSS scores and brain CT scan results were included in the analysis of sensitivity, specificity, positive predictive value (PPV), and negative predictive value (NPV) of both the individual components of the CPSS and the total CPSS, along with corresponding confidence intervals.

## Results

During the study period, a total of 7095 patients were triaged at the KATH ED. Of these, 116 presented with clinical features suggestive of stroke and were approached. A total of 110 patients consented and were recruited, comprising 51 males (46.4%) and 59 females (53.6%), with ages ranging from 33 to 92 years and a median of 54 years (IQR: 48-67). Ninety-five patients (86.37%) had received formal education, and 78 (70.91%) were employed. The majority of patients (89, 80.91%) were referred from primary care facilities, and the predominant mode of arrival was via private vehicle (58, 52.73%). After triaging with the SATS, 43 patients (39.09%) were assigned to the red category, indicating high urgency, while 67 patients (60.91%) were assigned to the orange category, indicating medium-level urgency.

Of the 110 patients interviewed, 99 (90%) underwent CT imaging, while 11 (10%) did not receive imaging, primarily due to financial constraints or because they died before CT imaging could be done. Sixty patients (54.6%) had their imaging done at KATH, while 39 patients (35.5%) had imaging before referral to KATH. Eighty-six patients (78.2%) had a diagnosis of stroke on CT scan (comprising 40 ischemic strokes and 46 hemorrhagic strokes), five (4.6%) had normal findings, and eight (7.3%) had alternative diagnoses (Table 1).

**Table 1:** Sociodemographic distribution, triage categorization, referral status, and imaging information of recruited patients. SATS: South African Triage Scale; KATH: Komfo Anokye Teaching Hospital; CT: computed tomography.

Variable	Frequency (N = 110)	Percentage
Age group		
<40 years	11	10.00
40-60 years	59	53.64
>60 years	40	36.36
Gender		
Male	51	46.36
Female	59	53.64
Educational level		
None	15	13.64
Primary	47	42.73
Secondary	37	33.64
Tertiary	11	10.00
Religion		
Christian	102	92.73
Muslim	8	7.27
Employment status		
Employed	78	70.91
Not employed	32	29.09
Triage (SATS) category		
Red	43	39.09
Orange	67	60.91
Yellow	0	0.00
Referral status		
Referred	89	80.91
Self-reported	21	19.09
Mode of arrival		
Ambulance	51	46.36
Private vehicle	58	52.73
Foot	1	0.91
CT scan done		
Yes	99	90.00
No	11	10.00
Source of CT scan image		
KATH	60	54.55
Referring hospital	39	35.45
CT scan not done	11	10.00
CT scan findings		
Stroke	86	78.18
Normal CT	5	4.55
Alternative diagnosis	8	7.27
CT scan not done	11	10.00

On reviewing the CPSS scores, 18 patients were excluded due to incomplete entries. The main reasons provided were that patients had a reduced level of consciousness and relatives were unable to provide reliable information. The remaining 92 patients with complete scores were analyzed. Among these patients, 35 (38%) scored 1 for facial droop, 72 (78.3%) scored 1 for arm drift, and 72 (78.3%) scored 1 for speech impairment. Overall, 87 patients (94.6%) had a total CPSS score ≥1 (Table 2).

**Table 2:** Distribution of CPSS results for patients with complete data. CPSS: Cincinnati Prehospital Stroke Scale.

CPSS assessments	Frequency (N = 92)	Percentage
Face deviation present = 1	35	38.04
Speech impairment present = 1	72	78.26
Arm drift present = 1	72	78.26
Total CPSS ≥1	87	94.57
Total CPSS = 0	5	5.44

**Figure 1:**
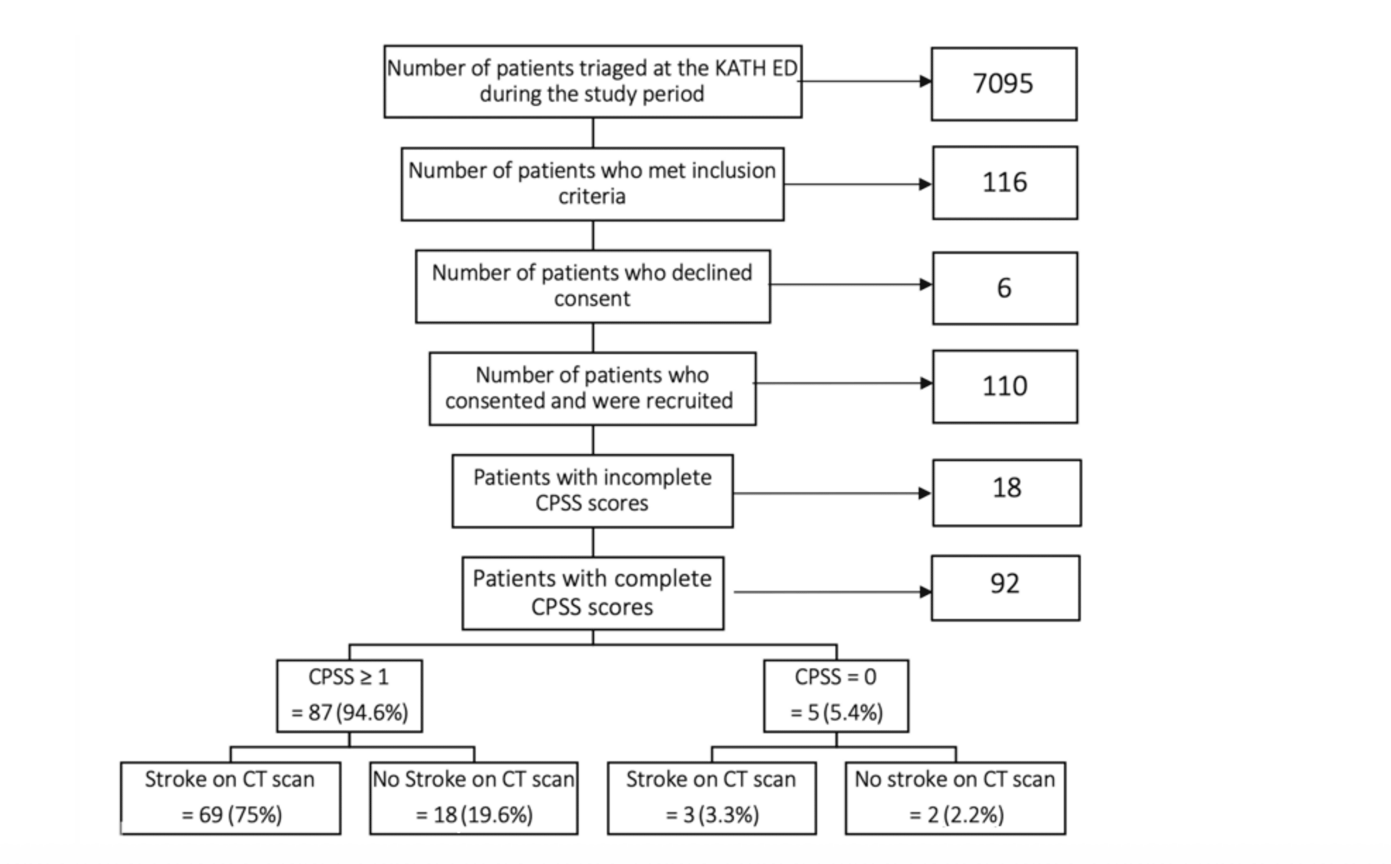
Flowchart of patient recruitment and how the CPSS results correspond with stroke diagnosis on CT scan. KATH: Komfo Anokye Teaching Hospital; ED: emergency department; CPSS: Cincinnati Prehospital Stroke Scale; CT: computed tomography.

All 92 patients with complete CPSS scores also had CT scan results available. Of these, 69 patients (75%) had a CPSS score ≥1 and also had confirmed stroke on CT scan, while 18 patients (19.6%) had a CPSS score ≥ 1 but no stroke on CT scan. Of the five patients (5.4%) who had a CPSS score of 0, three (3.3%) had confirmed stroke on CT scan, and two (2.2%) did not have stroke (Figure 1).

The CPSS had a sensitivity of 88.5% (CI: 69.8-97.6) and a specificity of 25.5% (CI: 15.8-38.0), a PPV of 31.9% (CI: 21.4-44.0), and a NPV of 85.0% (CI: 62.1-96.8). Individual sensitivities for the components of the CPSS were as follows: facial droop (82.9%, CI: 66.4-93.4), arm drift (81.9%, CI: 71.1-90.0), and speech impairment (79.2%, CI: 68.0-87.8). When the components were paired, facial droop + arm drift had the highest sensitivity (90.0%, CI: 73.5-97.0) (Table 3).

**Table 3:** Sensitivity, specificity, PPV, and NPV of the CPSS and its components. CI: confidence interval; PPV: positive predictive value; NPV: negative predictive value.

Variable	Sensitivity	CI	Specificity	CI	PPV	CI	NPV	CI
Face	82.9	66.4–93.4	24.6	14.1 – 37.8	40.3	28.9–52.5	70.0	45.7–88.1
Arm	81.9	71.1–90.0	35.0	15.4–59.2	81.9	71.1–90.0	35.0	15.4–59.2
Speech	79.2	68.0–87.8	25.0	8.7–49.1	79.2	68.0–87.8	25.0	8.7–49.1
Face + arm	90.0	73.5–97.0	27.4	16.9–40.2	37.5	26.4–40.2	85.0	62.1–96.8
Face + speech	83.3	65.3–94.4	24.2	14.2–36.7	34.7	23.9–46.9	75.0	50.9–91.3
Arm + speech	81.0	68.6–90.1	26.5	12.9–44.4	65.3	53.1–76.1	45.0	23.1–68.5
Face + arm + speech	88.5	69.8–97.6	25.8	15.8–38.0	31.9	21.4–44.0	85.0	62.1–96.8

## Discussion

The results of this study showed that the CPSS had a high sensitivity of 88.5% and a low specificity of 25.8% for identifying patients with stroke at the KATH ED. This suggests that the CPSS can be a good screening tool for stroke but would perform poorly as a confirmatory test in ruling out patients without the disease.

In 2009, a study conducted by Frendl et al. in the United States found that the CPSS had a high sensitivity of 94% and a low specificity of 20% for identifying strokes [11]. These results were consistent with those of our study; however, in their study, the CPSS assessments were conducted by trained paramedics in the prehospital setting, who were involved in patient transfers to two stroke care facilities.

Research by Zohrevandi et al. [12] in Iran in 2014 and De Luca et al. [13] in Italy in 2019 also reported high sensitivities of the CPSS for detecting stroke, although the specificities in both studies were higher, with values exceeding 50%. In the study conducted by Zohrevandi et al., retrospective data from emergency room admissions were used to calculate the CPSS scores, which were then compared to the final diagnosis by a neurologist. In the systematic review by De Luca et al., a diverse range of studies were examined, including those with CPSS assessments performed by physicians and paramedics.

A separate study by Maddali et al. analyzed the CPSS scores from trained Emergency Medical Technicians (EMTs) for patients with suspected stroke transported to a large stroke center in India. The results showed that the CPSS had a sensitivity of 81% for stroke, but notably, the specificity in this study was 100% [14]. 

The CPSS consistently demonstrated high sensitivities for identifying stroke in various studies; however, its specificity varied across different studies and clinical settings. This variation in specificity is likely due to the heterogeneity in the level of training and experience of the individuals conducting the assessment across these studies. Supporting this assumption, De Luca et al., in their study evaluating the role of the CPSS in detecting stroke in the ED, found no significant differences in sensitivity when the CPSS was administered by physicians and non-physicians. However, they observed that assessment by physicians resulted in higher specificity compared to those conducted by non-physicians [13].

Our study results further showed that the CPSS had a low PPV of 31.9% and a high NPV of 85% for stroke. This suggests that, when a CPSS score is 0, an individual is less likely to have a stroke. These findings also aligned with the results of Frendl et al.’s study but contrast with the findings of Zohrevandi et al.’s study [11,12]. It is important to note that, unlike sensitivity and specificity, the PPV and NPV values are significantly influenced by the prevalence of the disease and the sample size used in the calculations. Since our study involved a limited number of patients, particularly in the group with a CPSS score of 0, the accuracy and reliability of the PPV and NPV results in this study may have been impacted.

Maddali et al. [14] found a PPV of 100% in their study. Although a small sample size was also used in their study, the relative proportion of individuals with a CPSS score of 0 was higher, which provided a more balanced distribution of patients, allowing for a more accurate determination of both the PPV and NPV.

In our study, the sensitivities of the individual components of the CPSS for stroke did not differ much, but face deviation showed the highest sensitivity, consistent with the results of a study by Karimi et al. in Iran [15]. In contrast, Maddali et al. found that arm drift had the highest sensitivity, followed by face deviation [14].

Overall, the CPSS serves two crucial purposes. Firstly, it provides prehospital personnel with a quick and easy-to-use tool for rapid identification of patients who may be experiencing a stroke, thus facilitating prompt transfer to stroke-capable facilities. Secondly, it can help identify the subpopulation of individuals who are likely to benefit from thrombolysis and other acute stroke interventions in the emergency setting, thus allowing them to be prioritized for early imaging [7].

Our study findings validated the CPSS as an effective tool for screening strokes in triage situations. Given that a majority of stroke patients in our study were referred from lower-level health facilities, using the CPSS in the prehospital setting may be more beneficial, where it can aid in early identification of strokes at primary care, leading to early and appropriate referrals. Furthermore, training EMTs to use this tool for rapid identification of stroke symptoms during patient transportation, especially from their homes, can ensure timely transfer to stroke-capable facilities. The findings of this study can provide a foundation for targeted recommendations and effective implementation of the CPSS in prehospital stroke care in low-resource settings.

Neuroimaging for suspected stroke patients can be delayed in settings like ours for several reasons, including high service costs, staffing shortages, and ED overcrowding [16]. In this study, most patients received CT scan imaging after arriving at KATH. To minimize delays in neuroimaging and critical interventions, the CPSS can help identify likely stroke patients at triage and prioritize them for imaging. This is particularly important in busy and under-resourced areas in sub-Saharan Africa, where essential assessments and evaluations often face considerable delays.

Limitations

Some stroke signs were reported by patient relatives or eyewitnesses, particularly in cases where patients were unresponsive, which limits the reliability of the information. While the use of CT scans for confirming stroke diagnosis was standard of care at this facility, it is less accurate and sensitive for detecting strokes compared to more advanced imaging modalities, with a sensitivity of only 39-53% and specificity of 80% [17]. As a result, some stroke diagnoses may have been missed, which could have impacted our study results.

Additionally, some patients or their relatives may have been aware of the diagnosis before arrival, especially those who had already undergone CT imaging. Although this awareness should not inherently affect the CPSS assessment if performed correctly, it could have led triage nurses to score these patients more favorably, thus reducing reliability and potentially contributing to the high sensitivity and the low PPV observed.

Furthermore, this study used a limited sample size from a single site, which restricts the generalizability of our findings. Additionally, a significant number of patients were excluded due to incomplete CPSS entries or the inability to obtain CT imaging and were not accounted for in the sensitivity and specificity analysis. Finally, the small number of patients with a CPSS score of 0, compared to those with a CPSS score of 1 or higher, created a skewed distribution, which could have also impacted the results of the PPV and NPV analyses.

## Conclusions

The CPSS, administered by trained triage nurses, demonstrated a high sensitivity for detecting patients with stroke in the KATH ED, although its specificity was low. Facial droop was the most sensitive component of the CPSS. However, brain scans are still required to confirm the diagnosis and determine the stroke subtype. The CPSS is an easy-to-use screening tool whose implementation in emergency prehospital systems and protocols is highly recommended. Future studies should explore its feasibility and adaptation across a wider group of prehospital personnel, including EMTs in Ghana
